# Unveiling the impact of corticosteroid therapy on liquid biopsy-detected cell-free DNA levels in meningioma and glioblastoma patients

**DOI:** 10.1016/j.jlb.2024.100149

**Published:** 2024-03-20

**Authors:** Veronica Aran, Jose Orlando de Melo Junior, Carlos Pilotto Heming, Daniel Jaime Zeitune, Vivaldo Moura Neto, Paulo Niemeyer Filho

**Affiliations:** Instituto Estadual do Cérebro Paulo Niemeyer (IECPN), R. do Rezende, 156 – Centro, Rio de Janeiro, 20231-092, Brazil

**Keywords:** Liquid biopsy, cfDNA, Intracranial tumors, Glioblastoma, Meningioma

## Abstract

The liquid biopsy era has brought several possibilities to improve precision in patient care. Among the different sources of analytes, the cfDNA has been explored as a possible disease indicator, especially in cancer. Intracranial tumors still represent a challenge for liquid biopsy due to the blood–brain barrier being able to restrain both the migrating tumor cells and the liberation of cfDNA into the blood circulation. The aim of this work was to compare the differences between the cfDNA concentration in the plasma from patients with central nervous system tumors, and for this we analyzed a cohort of 188 individuals with glioblastoma (N = 57), brain metastasis (N = 15), meningioma (N = 90) and schwannoma (N = 26). Plasma samples were obtained immediately before tumor excision, and the cfDNA was isolated from the samples and quantified. The results showed that cfDNA plasma levels vary according to the tumors analyzed, with glioblastoma and brain metastasis presenting higher median levels of cfDNA than meningiomas and schwannomas. In addition, corticosteroid treatment resulted in higher cfDNA levels in meningioma and glioblastoma patients and vasogenic brain edema resulted in higher cfDNA levels only in meningioma patients. We hypothesize that cfDNA evaluation might have clinical monitoring value and that other clinical variables, such as corticosteroid used, should be considered during the liquid biopsy clinical evaluation of intracranial tumors.

## 1 Introduction

Liquid biopsy represents a minimally invasive procedure, which can be useful to generate information on disease status such as cancer. There are different sources for liquid biopsy and examples include urine, cerebrospinal fluid, saliva and blood, which may contain different molecules such as cell free DNA (cfDNA), circulating tumor DNA (ctDNA), circulating tumor cells (CTCs), miRNA, RNA, extracellular vesicles (EVs), etc [1]. Analyzing these molecules can prove invaluable; for instance, variations in cfDNA levels have shown associations with various clinical factors such as inflammatory diseases, cancer, trauma, exercise, and cardiovascular conditions. This suggests that cfDNA has the potential to serve as a biomarker with diagnostic, prognostic, and predictive applications [2]. cfDNA is released into the circulation via different cellular events such as necrosis, apoptosis, and active secretion [3]. Patients with cancer usually show higher levels of cfDNA in their blood compared to healthy individuals, since tumor cells divide more rapidly than normal cells, consequently releasing more cfDNA in the bloodstream [4].

There are different types of cfDNA including ctDNA, foetal DNA in pregnant women and donor-derived DNA in transplant recipients. Longitudinal monitoring of circulating DNA has attracted much attention, owing to the minimal invasiveness of sample collection and short half-life of cfDNA. Interestingly, quantitative studies demonstrated that cfDNA concentrations in the blood of cancer patients averages six times the concentration found in healthy individuals [1]. A similar pattern was noticed in patients with advanced cancer when compared to early-stage cancer patients, which often present lower ctDNA concentrations [5]. In addition, high levels of cfDNA are often related to rapid deterioration, whereas low cfDNA expression can be encountered in patients with improved clinical symptoms [6].

Comparisons of cfDNA and ctDNA have been conducted for some cancer types. A comparison between the levels of cfDNA and ctDNA in hepatocellular cancer patients were accessed to evaluate therapy response and clinical outcome indicating that individuals who presented metastasis had significantly higher amounts of plasmatic cfDNA than those who did not [7], a finding that was similarly observed in other tumor types such as oral squamous cell carcinoma [8] and breast cancer [3,9]. Interestingly, patients with hepatocellular cancer-associated with hepatitis C virus with tumor sizes of over 5 cm and vascular invasion showed high levels of total cfDNA in the bloodstream [10]. Dynamic changes on cfDNA levels have also been monitored following surgical tumor removal, where the concentration of cfDNA decreased in breast [9] and liver cancers [11], which could point to cfDNA levels being indicative of surgery efficacy. Furthermore, cfDNA trends could be paralleled with patients’ clinical history and drug treatment becoming a potential biomarker for therapy response [7].

There are not many studies on this subject in central nervous system (CNS) tumors, however a study from 2022 revealed that diffuse glioma patients who did not respond to radiation therapy exhibited significantly higher pre-radiotherapy amounts of cfDNA than those who did [12]. In fact, glioblastoma patients have been associated with detectable cfDNA concentrations in plasma, which could possible occur due to the blood-brain barrier disruption. Besides, a recent published study reported that cerebrospinal fluid from glioma patients contained higher cfDNA concentrations close to the tumor location than in the CSF collected from lumbar puncture, and that cfDNA could be a good diagnostic tool in the molecular profiling of patients in order to stratify them according to prognostic and treatment subgroups [13]. However, little is known regarding the comparisons of plasma cfDNA among different types of CNS tumors.

While the number of actionable molecular alterations for targeted therapies is limited in patients with CNS tumors, and there is no current FDA-approved test for liquid biopsy for these tumors, the prospect of identifying them non-invasively is still attractive by using techniques which include isolating CTCs for genetic analysis, detecting mutations or methylation in cfDNA, and examining EV cargo [14]. Furthermore, the utility of methylation analysis in brain tumor diagnostics was exemplified by the creation of a DNA methylation-based classification system for CNS tumors using a machine learning approach. Advanced methylation-based sequencing enabled the identification of distinct methylation patterns and precise classification of brain tumors using circulating cfDNA, which may be considered a promising tool for liquid biopsy analysis of CNS tumors [14,15].

Considering the lack of enough data regarding on intracranial tumors’ liquid biopsy and cfDNA status, our aim was to investigate the plasma levels of cfDNA in patients with meningioma, schwannoma, brain metastasis and glioblastoma before tumor resection, and to correlate the results with the clinicopathological characteristics of the patients.

## 2 Materials and methods

### 2.1 Study population

The study included 188 patients diagnosed with four different tumor types: glioblastoma, brain metastasis, meningioma and schwannoma. The eligible cases included adult patients who were diagnosed with the aforementioned tumors between 2021 and 2022 at the *Instituto Estadual do Cérebro Paulo Niemeyer (IECPN,* Rio de Janeiro. Patients' data, including clinical characteristics such as sex (based on chromosomal genotype), age at diagnosis, tumor histology, and treatments undertaken; were collected from their medical records. Written informed consent was obtained from all patients and the present study was approved by the local Human Ethics Committee of the *Instituto Estadual do Cérebro Paulo Niemeyer* (protocol N^o^ CAAE 90680218.6.0000.8110). Inclusion criteria consisted of: (1) patient with confirmed histopathological diagnosis of glioblastoma, brain metastasis, meningioma, or schwannoma; and (2) Patients’ age had to be at least 18 years. Exclusion criteria included: (1) absence of histopathological confirmation of the tumor type; and (2) inadequate material for analysis.

### 2.2 Samples and cfDNA extractions

Liquid biopsy was performed in order to obtain the patients' samples. The patient's peripheral blood was collected via venipuncture immediately before the surgery, obtaining a total of 8 mL of blood using EDTA tubes. The blood was taken to the laboratory where the plasma was separated from the serum by two consecutive centrifugations (the first 1,200×*g* ​for 10 min and the second at 16,000×*g* ​for 15 min at 4 °C). In order to extract cfDNA from the plasma, the plasma samples were transferred to the Maxwell® RSC automated Instrument (Promega Corporation, USA) using the manufacturer's protocols and kits. The kit chosen was the Maxwell RSC ccfDNA plasma kit (Promega), which utilizes an innovative paramagnetic particle to purify circulating DNA, which enhances sample capture, washing, and purification of circulating DNA. The instrument uses magnetic particle-handling devices that effectively bind circulating DNA to the paramagnetic particle in the initial well of a preloaded cartridge and facilitate mixing during processing.

The eluted cfDNA samples (1 μl cfDNA) were quantified using Quantus™ Fluorometer (Promega Corporation, USA), which is intended for compatibility with Promega QuantiFluor® Dye Systems (in our case, QuantiFluor® One dsDNA system), allowing precise fluorescence-based quantification of dsDNA, ssDNA, and RNA. A dedicated protocol for each QuantiFluor® Dye is provided on the machine's screen, with optimized calibration curve settings tailored to blank, standard, sample, and dye preparation steps. Good quality samples are read, and in case of low abundance, or poor quality, no measurement is achieved. The results are obtained in μg/μl.

### 2.3 Statistical analysis

Standard qualitative and quantitative statistical analyses were conducted with Prism 9.0 software (GraphPad Software, Inc.). Chi-square (X_2_) and Fisher's exact tests were used for comparison between categorical variables as appropriate. Mann-Whitney test was used for ordinal qualitative and quantitative variables with non-normally distributed data. Student's t-test was used for quantitative variables with normally distributed data. Correlation analyses were performed using the Pearson correlation method for normally distributed data and the Spearman correlation for non-normally distributed data. Kruskall-Wallis and multiple comparisons tests was used to compare cfDNA concentration between the groups of pathologies. The Kolmogorov-Smirnov normality test was used to verify the distribution of the data of all continuous variables. A p-value <0.05 was considered statistically significant.

## 3 Results

### 3.1 Cohort characteristics

The study included 188 plasma samples from patients with glioblastoma (N = 57), brain metastasis (N = 15), meningioma (N = 90) and schwannoma (N = 26). The characteristics of the study population are shown in Table 1. Patients mean age was 60 years (21–80) for glioblastoma, 62 years (43–74) for brain metastasis, 58 years (20–81) for meningioma, and 51 years (23–73) for schwannoma. Median plasma cfDNA concentration in ng/μl was 0.4 (0.17–0.72) for glioblastoma, 0.44 (0.21–0.80) for brain metastasis, 0.15 (0.08–0.34) for meningioma, and 0.11 (0.08–0.32) for schwannoma. Epilepsy was present in 35% of Glioblastoma, 13% of brain metastasis, 32% of meningioma and no cases were observed in the schwannoma patients. Most patients underwent treatment with the corticosteroid, where meningioma and schwannoma presented less patients on this treatment (29% and 23%, respectively) compared to glioblastoma and brain metastasis (89% and 100%, respectively). Vasogenic brain edema was present in all glioblastoma and brain metastasis tumors, while present in 60% of meningioma and absent in schwannoma. The mean tumor volume was higher in glioblastoma and brain metastasis compared to meningioma and schwannoma. Clinicopathological characteristics are summarized in Table 1.

**Table 1:** Overall characteristics of the patient cohort grouped by histopathology.

Histopathology	Glioblastoma	Metastasis	Meningioma	Schwannoma
**No of patients**	57	15	90	26
**Sex (male/female)**	22 (39%)/35 (61%)	6 (40%)/9 (60%)	24 (27%)/66 (73%)	16 (62%)/10 (38%)
**Mean age, yrs. (range)**	60 (21–80)	62 (43–74)	58 (20–81)	51 (23–73)
**Epilepsy**	20 (35%)	2 (13%)	29 (32%)	0 (0%)
**Corticosteoid use**	51 (89%)	15 (100%)	26 (29%)	6 (23%)
**Vasogenic brain edema**	57 (100%)	15 (100%)	54 (60%)	0 (0%)
**Mean tumor volume cm**^**3**^**(range)**	61.13 (9.67–183.1)	34.40 (3.03–89.6)	29.84 (0.84–210.52)	16.43 (0.23–58.65)
**Median plasma cfDNA concentration (ng/μl)**	0.4 (0.17–0.72)	0.44 (0.21–0.80)	0.15 (0.08–0.34)	0.11 (0.08–0.32)

### 3.2 cfDNA concentration varies depending on the tumor analyzed

**Fig. 1:**
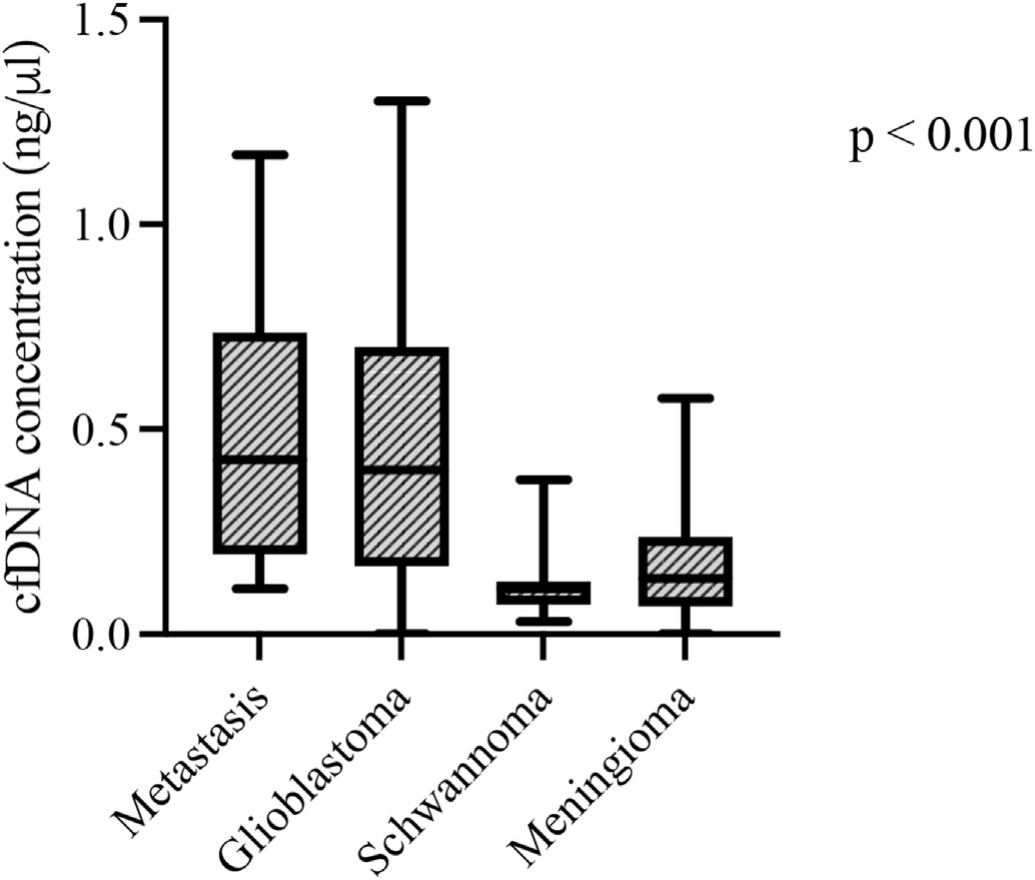
Boxplot of plasma cfDNA concentration (ng/μl) and tumor types. Boxes extend from the 25th to the 75th percentile of each group's distribution of values; the central horizontal line within the boxes denote median value and vertical extending lines denote adjacent values. The plasma cfDNA concentration was statistically different between the groups of tumors (p < 0.001), with higher levels in metastasis and glioblastoma.

Levels of cfDNA were measured in each of the 188 plasma samples. The median level in each group of tumors was 0.4 ng/μl (0.17–0.72 ng/μl) in glioblastoma, 0.44 ng/μl (0.21–0.80 ng/μl) in brain metastasis, 0.15 ng/μl (0.08–0.34 ng/μl) in meningioma, and 0.11 ng/μl (0.08–0.32 ng/μl) in schwannoma revealing a significant difference (p < 0.001) in cfDNA concentration among them (Table 1 and Fig. 1). Brain metastasis and Glioblastoma showed higher levels of cfDNA when compared to Schwannoma and Meningioma (Fig. 1). Multiple comparison's test for plasma cfDNA concentration between the tumors indicated statistically significant higher levels of cfDNA in glioblastoma and brain metastasis when compared to meningioma and schwannoma (Fig. 1). Difference was also significant observed on distribution patterns with a greater spread of values in glioblastoma and metastasis.

#### 3.2.1 The concentration of cfDNA is higher in meningioma patients taking corticosteroid or with vasogenic brain edema

The levels of cfDNA were analyzed against different variables such as age, sex, history of epilepsy, history of radiotherapy, corticosteroid use, histologic graduation (WHO), Ki-67 proliferative index, histological mitotic number, brain infiltration, brain edema, tumor border, and tumor volume. We found that the median plasma cfDNA concentration was significantly higher in the presence of vasogenic brain edema in meningioma patients (p = 0.04) (Table 2). In addition, we observed that patients under corticosteroid use showed significant increase in plasma cfDNA levels (p = 0.01) (Table 2). Sex, history of epilepsy, history of radiotherapy, histologic graduation (WHO), brain infiltration, brain edema, tumor border was not statistically associated with different levels of plasma cfDNA (Table 2). Age, Ki-67 proliferative index, histological mitotic number and tumor volume was not correlated with concentrations of plasma cfDNA (p = 0.35, p = 0.90, p = 0.47 and p = 0.42, respectively).

**Table 2:** Analysis of cfDNA levels compared to different variables in meningioma.

Variables	Plasma cfDNA concentration	*p*-value
**Sex**		0.86
Male	0.14 (0.09–0.27)	
Female	0.15 (0.06–0.35)	
**History of epilepsy**		0.19
No	0.14 (0.08–0.31)	
Yes	0.21 (0.08–0.42)	
**History of radiotherapy**		0.13
No	0.16 (0.07–0.35)	
Yes	0.11 (0.03–0.20)	
**Corticosteroid use**		0.01
No	0.12 (0.05–0.21)	
Yes	0.23 (0.09–0.43)	
**Histologic graduation (WHO)**		0.20
Grade I	0.14 (0.07–0.31)	
Grade II	0.21 (0.10–0.39)	
**Brain infiltration on histopathology**		0.27
No	0.14 (0.07–0.32)	
Yes	0.22 (0.09–0.37)	
**Brain edema on MRI scan**		0.04
No	0.12 (0.03–0.23)	
Yes	0.19 (0.09–0.38)	
**Tumor border on MRI scan**		0.90
Regular	0.17 (0.07–0.36)	
Irregular	0.15 (0.09–0.32)	

#### 3.2.2 The concentration of cfDNA is higher in glioblastoma patients taking corticosteroid

The levels of cfDNA were evaluated taking in consideration age, sex, corticosteroid use, history of epilepsy, history of radiotherapy, tumor affecting both hemispheres (bilateral tumor), reoperation for recurrent tumor and tumor volume. In our series, all glioblastoma patients had vasogenic brain edema, brain infiltration and irregular border. Therefore, comparisons using these variables were not possible to compute. Furthermore, all glioblastoma is classified as grade IV (World Health Organization – WHO [16]), and Ki-67 proliferative index and histological mitotic number are virtually high in this pathology and is not routinely checked in our institution. Thus, these variables were not evaluated in our glioblastoma series.

Plasma concentration of cfDNA was statistically higher in patients under corticosteroid use (p = 0.049) (Table 3). Other variables such as sex, history of epilepsy, history of radiotherapy, tumor affecting both hemispheres and reoperation for recurrent tumor were not statistically associated with different levels of plasma cfDNA (Table 3). Age and tumor volume were not correlated with concentrations of plasma cfDNA (p = 0.20 and p = 0.53, respectively).

**Table 3:** Analysis of cfDNA levels compared to different variables in glioblastoma.

Variables	Plasma cfDNA concentration (ng/μl)	p-value
**Sex**		0.27
Male	0.36 (0.16–0.65)	
Female	0.46 (0.23–0.84)	
**Corticosteroid use**		0.049
No	0.19 (0.15–0.29)	
Yes	0.42 (0.19–0.77)	
**History of epilepsy**		0.11
No	0.35 (0.14–0.61)	
Yes	0.54 (0.20–1.00)	
**History of radiotherapy**		0.68
No	0.40 (0.19–0.71)	
Yes	0.35 (0.13–0.81)	
**Bilateral tumor**		0.51
No	0.30 (0.16–0.62)	
Yes	0.40 (0.19–0.73)	
**Reoperation for recurrent tumor**		0.79
No	0.39 (0.18–0.69)	
Yes	0.51 (0.13–0.87)	

#### 3.2.3 Schwannoma

The levels of cfDNA were also analyzed against sex, corticosteroid use, history of radiotherapy, and tumor volume. Neither of patients had vasogenic brain edema, nor irregular borders. Therefore, comparisons of the vasogenic brain edema impact on cfDNA plasma levels were not possible to compute. Table 4 shows that sex, corticosteroid use, and history of radiotherapy was not associated with different measures of concentration of plasma level cfDNA (p = 0.88, p = 0.36 and p = 0.87, respectively). Furthermore, tumor volume was not correlated with concentrations of plasma cfDNA (p = 0.24). Other variables such as irregular borders were not evaluated due to the restricted number of cases to be computed by statistic tool.

**Table 4:** Analysis of cfDNA levels compared to different variables in schwannoma.

Variables	Plasma cfDNA concentration (ng/μl)	*p*-value
**Sex**		0.88
Male	0.11 (0.07–0.24)	
Female	0.11 (0.05–0.13)	
**Corticosteroid use**		0.36
No	0.11 (0.07–0.12)	
Yes	0.14 (0.06–0.35)	
**History of radiotherapy**		0.87
No	0.11 (0.05–0.13)	
Yes	0.11 (0.07–0.20)	

#### 3.2.4 Corticosteroid use is a variable linked to higher levels of cfDNA in meningioma and glioblastoma

**Fig. 2:**
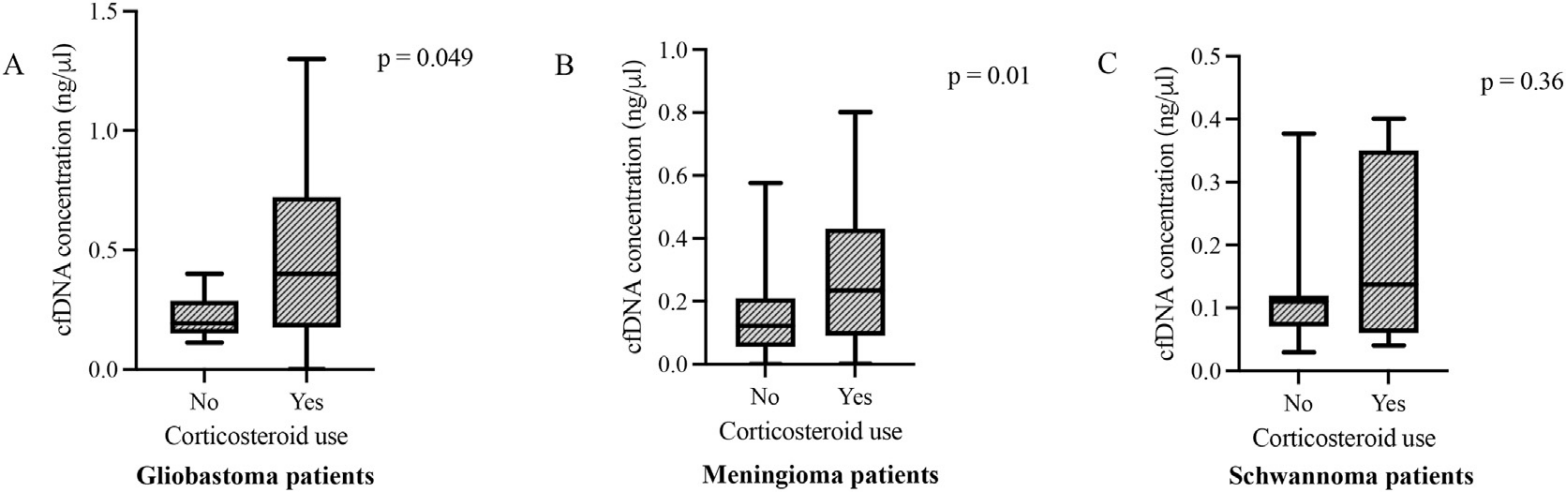
Boxplots of plasma cfDNA concentration and corticosteroid use in glioblastoma, meningioma and schwannoma patients. Boxes extend from the 25th to the 75th percentile of each group's distribution of values; the central horizontal line within the boxes denote median value and vertical extending lines denote adjacent values. (A) Plasma levels of cfDNA concentration was statistical higher in patients under corticosteroid use (p = 0.049). (B) There is statistically higher levels of plasma cfDNA in meningioma patients using corticosteroid compared to patients that do not take the medication (p = 0.01). (C) The schwannoma group presented no statistical difference in cfDNA plasma levels between presence and absence of corticosteroid (p = 0.36).

In the present analysis, we compared the concentrations of cfDNA in the presence and absence of corticosteroid use in glioblastoma, meningioma and schwannoma (Fig. 2). Brain metastasis was not included since all the patients in this group underwent corticosteroid therapy. Glioblastoma and meningioma showed statistical higher levels of cfDNA in the presence of corticosteroid use, medians of 0.42 ng/μl (0.19–0.77 ng/μl) and 0.23 ng/μl (0.09–0.43 ng/μl), when compared to corticosteroid absence, medians of 0.19 ng/μl (0.15–0.29 ng/μl) and 0.12 ng/μl (0.05–0.21 ng/μl), respectively (p = 0.049 and p = 0.01, respectively) (Fig. 2 A and 2B). The schwannoma group showed no statistical difference in cfDNA plasma levels between corticosteroid use, median of 0.14 ng/μl (0.06–0.35 ng/μl), and corticosteroid absence, median of 0.11 ng/μl (0.07–0.12 ng/μl), p = 0.36 (Fig. 2C).

#### 3.2.5 Vasogenic brain edema is a variable linked to higher levels of cfDNA independently of corticosteroid use

**Fig. 3:**
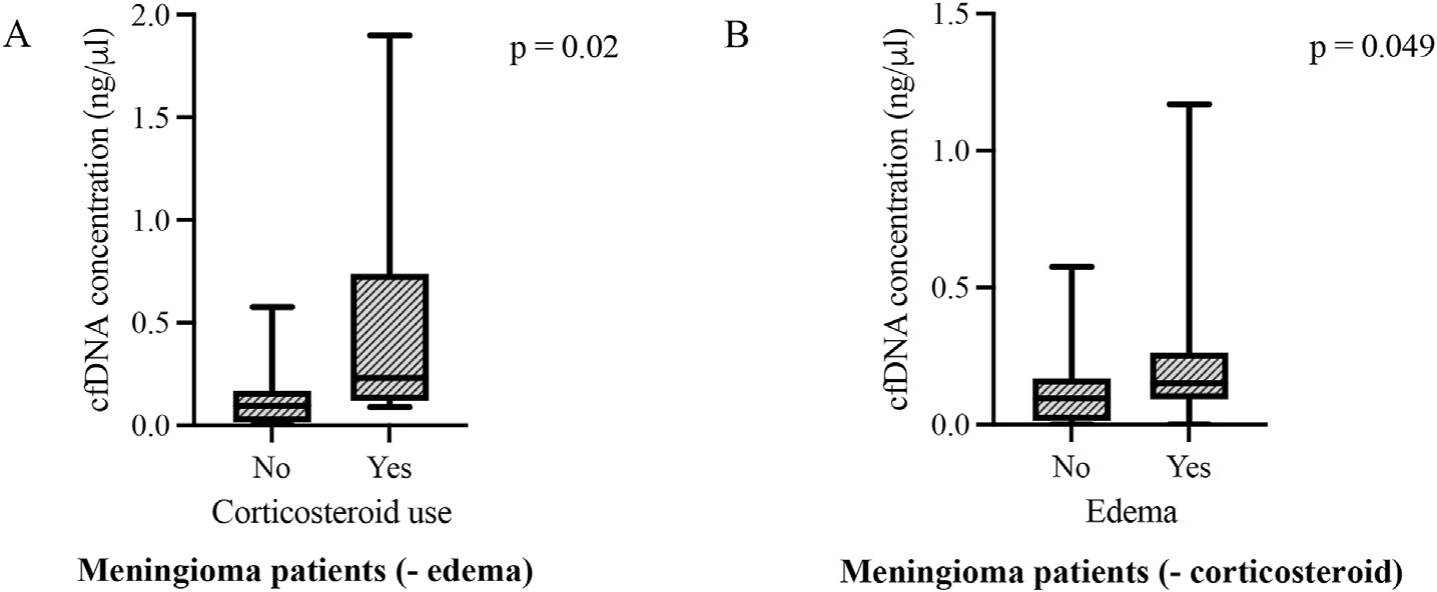
Boxplots of plasma cfDNA concentration in the presence of corticosteroid only or edema only in meningioma patients. Boxes extend from the 25th to the 75th percentile of each group's distribution of values; the central horizontal line within the boxes denote median value and vertical extending lines denote adjacent values. (A) The plasma levels of cfDNA concentration were statistical higher in patients without edema (-edema) under corticosteroid use (p = 0.02). (B) The plasma levels of cfDNA concentration were significantly higher in meningioma patients with edema without corticosteroid (-corticosteroid), (p = 0.049).

In the clinical setting, corticosteroid use is usually recommended for patients with vasogenic brain edema. Thus, we compared if this variable could be responsible for the higher levels of cfDNA seen in meningioma with edema. Interestingly, in our meningioma series, the corticosteroid therapy was not found to be linked with the higher levels of cfDNA in patients with brain edema versus patients without brain edema (34% vs 25%; p = 0.13, data not shown). When variables were set apart, the vasogenic brain edema and corticosteroid use were independently associated with higher levels of cfDNA (p = 0.049 and p = 0.02, respectively, Fig. 3).

## 4 Discussion

The analysis of blood derived cfDNA is not trivial due to cfDNA heterogeneity, low abundance and fluctuation in vivo [17]. In addition, cfDNA release in the bloodstream can occur due to different conditions apart from cancer such as reported in autoimmune diseases [18], patients with brain trauma [19] and cardiovascular diseases [20]. Thus, in the present study we analysed different CNS tumors in relation to cfDNA levels and other clinical variables.

Our data shows that the cfDNA plasma concentration varies depending on the tumor analyzed. Brain metastasis and glioblastoma showed significantly higher levels of plasma cfDNA when compared to schwannoma and meningioma (p < 0.001). In addition, an apparent correlation of corticosteroid treatment and elevated plasmatic cfDNA release in both meningioma and glioblastoma patients occurred, whereas for schwannoma patients the same analysis lacked significancy. Interestingly, another study also reported an increase in cfDNA in glioblastoma patients treated with corticosteroid therapy [21]. It is important to point out that the brain metastasis cfDNA levels was no included in the test against corticosteroid use since all the patients in this group underwent corticosteroid therapy. Corticosteroids are a class of steroid hormones that have been linked to contribute to apoptosis in different tissues. Since the CNS is vascularized, and contains pericytes, scientists isolated and cultured them from rat micro vessels, and observed that dexamethasone-induced apoptosis in these cells, an effect that was antagonized by a glucocorticoid antagonist termed RU486 [22]. In addition, other body cells were shown to undergo apoptosis in response to corticosteroids [23]. These could be one of the causes of the increased cfDNA levels observed upon corticosteroids treatment in some of the meningioma and glioblastoma patients included in the present study.

Although the specific impact of corticosteroid therapy on the levels of cfDNA in intracranial tumors remains to be determined, one could argue that corticosteroid use might modulate the permeability of the blood-brain barrier (BBB). The latter is a dynamic physical barrier comprising endothelial cells which can protect the brain from different compounds, with the aid of specific transporters termed ABC transporters [24]. High-dose corticosteroid therapy has been previously associated with elevated cfDNA levels in the serum of patients with systemic lupus erythematosus (SLE) during acute exacerbation of their disease [25]. Furthermore, in a proof-of-concept study by Magnusson and colleagues involving lung transplantation patients, one showed elevated levels of cfDNA in the serum following treatment with corticosteroids [26]. Interestingly, other study showed that corticosteroid therapy improves the tightness of the BBB via glucocorticoid receptors in health individuals [27]. However, it is well known that certain diseases and brain neoplasms can induce disintegration of proteins such as integral membrane proteins that form tight junctions allowing the breakage of the BBB [27] which may result in higher levels of cfDNA in the bloodstream that may explain the levels observed in glioblastoma, which is the most aggressive tumor type analysed, whereas in schwannomas and meningiomas the tumors occur outside the parenchyma, and are usually less aggressive types.

Another interesting result was that vasogenic brain edema represented a variable linked to higher levels of cfDNA independently of corticosteroid use, meaning they work separately in the process. Since vasogenic brain edema is known to cause inflammation and inflammation processes have been linked to high levels of cfDNA [28], we cannot guarantee that the cause is one or the other.

The higher levels of cfDNA in brain metastasis observed in our group of patients may be explained by both the reminiscent tumor cells from the primary tumor and the ability of these cells to metastasize to the brain [29], leading to a higher tumors cell burden. Furthermore, the brain metastasis group were also analysed in relation to different variables such as sex, multiple lesions, and tumor volume, but not vasogenic brain edema or cortiscosteroid use since all patients in this group presented vasogenic brain edema and underwent corticosteroid therapy. Therefore, comparisons of the impact of vasogenic brain edema and corticosteroid use on plasma levels of cfDNA were not possible to compute in brain metastasis. Sex and multiple lesions were not found to be associated with different levels of plasma cfDNA (p = 0.95 and p = 0.38, respectively). In addition, tumor volume was not correlated with concentrations of plasma cfDNA (p = 0.83). Other variables such as history of epilepsy was not evaluated due to the restricted number of cases to be computed by statistic tool.

Conventional correlation analysis methods fall short in capturing the intricate connections between biomarkers and the diverse nature of cancer subtypes. To address this limitation, researchers have proposed to employ machine learning techniques with liquid biopsy data to jointly explore the fundamental aspects of tumor biology [30]. A recent systematic review that analysed more than 400 papers, have observed that the early detection of cancer, through liquid biopsy, has been approached using diverse machine learning algorithms [30]. These algorithms have been tested across various cancer types, including pancreatic cancer, hepatocellular carcinoma, breast cancer, oral cancer, among others, targeting a broad spectrum of components, including cfDNA [30]. However, there are not much information available on brain tumors and diagnostic algorithms for CNS patients [31]. Nevertheless, utilizing critical characteristics found in liquid biopsy samples, such as the levels of cfDNA discussed in the present study, and evaluating it using a diagnostic algorithm for CNS patients could facilitate straightforward and non-invasive diagnosis and monitoring.

The present work indicates that cfDNA holds promise as a potential monitoring biomarker in high-grade gliomas. It's worth noting that the use of corticosteroids could introduce a misleading factor, potentially resulting in increased release of cfDNA independently of the tumor. The main limitation of considering cfDNA measures alone as a tumor biomarker is its lack of specificity since it might be elevated in other diseases such as cardiovascular diseases, in viral and bacterial infections or inflammation [32,33]. However, its measure associated with other findings might give important information on disease monitoring, especially after treatments such as chemotherapy and surgery [34]. It is important to point out that this study analysed cfDNA as a general measurable option common to all the tumors analysed, whereas ctDNA analysis would be more specific and ctDNA mutations would frequently differ among the different tumor types, which was not the objective of the present study.

## 5 Conclusion

In summary, the present data supports that plasma cfDNA levels exhibit variations depending on the type of CNS tumor analysed, with the more aggressive forms, such as glioblastoma and brain metastasis, demonstrating elevated cfDNA levels in contrast to the less aggressive types like meningioma and schwannoma. In addition, for the first time, we show that corticosteroid use increases the concentration of cfDNA in meningioma and glioblastoma patients. Despite the reasons for that being uncertain, one explanation could rely on increased tumor-derived cfDNA due to corticosteroid effects on cell death and turnover. In conclusion, cfDNA could hold potential in the clinical assessment of intracranial tumors. However, for a more accurate diagnosis it's crucial to take into account the medications being used, such as corticosteroids, in addition to other health conditions when assessing cfDNA levels.

## Ethics approval and consent to participate

Written informed consent was obtained from all patients and the present study was approved by the local Human Ethics Committee of the *Instituto Estadual do Cérebro Paulo Niemeyer* (protocol N^o^ CAAE 90680218.6.0000.8110).

## Contribution statement

VA: Conceptualization, Methodology, Data curation, Writing-original draft, Investigation, Supervision, Writing – review & editing. JOML: data curation, statistical analysis, clinical data acquisition, Writing – original draft, review & editing; CPH: Writing – review & editing; DJZ: clinical data acquisition; VMN and PNF: Funding acquisition and critically reviewed the manuscript. All authors participated in the preparation of this manuscript and have read and approved its final draft for publication.

## Funding

This study was funded by the Brazilian agency *Fundação de Amparo à Pesquisa doEstado doRio de Janeiro* *(**FAPERJ 25191**).* The funding agency had no role in the analysis, development, interpretation of the data or decision to submit the manuscript.

## Declaration of competing interest

The authors declare that they have no known competing financial interests or personal relationships that could have appeared to influence the work reported in this paper.
